# Copy number variation at the 22q11.2 locus influences prevalence, severity, and psychiatric impact of sleep disturbance

**DOI:** 10.1186/s11689-022-09450-0

**Published:** 2022-07-10

**Authors:** Kathleen P. O’Hora, Amy Lin, Leila Kushan-Wells, Carrie E. Bearden

**Affiliations:** 1 Department of Psychiatry and Biobehavioral Sciences, Semel Institute for Neuroscience and Human Behavior, University of California, A7-460 Semel Institute, Los Angeles, CA 90095 USA; 2 Neuroscience Interdepartmental Program, University of California, Los Angeles, CA USA; 3 Department of Psychology, University of California, Los Angeles, CA USA

**Keywords:** Sleep, Psychosis, Schizophrenia, Psychosis-risk symptoms, Autism spectrum disorder, 22q11.2 locus, Copy number variation, Developmental neuropsychiatric disorders, Executive function, Behavioral problems

## Abstract

**Background:**

Sleep disturbance is common, impairing, and may affect symptomatology in developmental neuropsychiatric disorders. Here, we take a genetics-first approach to study the complex role of sleep in psychopathology. Specifically, we examine severity of sleep disturbance in individuals with a reciprocal copy number variant (CNV) at the 22q11.2 locus and determine sleep’s effect on psychiatric symptoms. CNVs (deletion or duplication) at this locus confer some of the greatest known risks of neuropsychiatric disorders; recent studies suggest the 22q11.2 deletion negatively impacts sleep, but sleep disruption associated with 22q11.2 duplication has not been investigated.

**Methods:**

We compared subjective sleep disturbance and its relationship to psychiatric symptoms cross-sectionally and longitudinally over 1 year in 107 22q11.2 deletion (22qDel) carriers (14.56±8.0 years; 50% male), 42 22q11.2 duplication (22qDup) carriers (16.26±13.1 years; 54.8% male), and 88 age- and sex-matched controls (14.65±7.4 years; 47.1% male). Linear mixed models were used to compare sleep disturbance, assessed via the Structured Interview for Psychosis-Risk Syndromes (SIPS), across groups. Next, CNV carriers were categorized as good or poor sleepers to investigate sleep effects on multiple neurobehavioral traits: psychosis-risk symptoms (SIPS), autism-related behaviors (Repetitive Behavior Scale (RBS) and Social Responsiveness Scale (SRS)), real-world executive function (Behavior Rating Inventory of Executive Function (BRIEF)), and emotional/behavioral problems (Child Behavior Checklist (CBCL)). Linear mixed models tested the effect of sleep category and a group-by-sleep interaction on each measure, cross-sectionally and longitudinally.

**Results:**

22qDel and 22qDup carriers both reported poorer sleep than controls, but did not differ from each other. Cross-sectionally and longitudinally, poor sleepers scored higher on positive symptoms, anxious/depressed, somatic complaints, thought problems, and aggressive behavior, as well as RBS and SRS total scores. There were significant group-by-sleep interactions for positive symptoms and the majority of CBCL subdomains, in which the difference between good and poor sleepers was larger in 22qDel compared to 22qDup.

**Conclusions:**

Our findings indicate that CNVs at the 22q11.2 locus impact sleep which, in turn, influences psychopathology. Sleep disturbances can differentially impact psychopathology, depending on 22q11.2 gene dosage. Our findings serve as a starting point for exploring a genetic basis for sleep disturbance in developmental neuropsychiatric disorders.

**Supplementary Information:**

The online version contains supplementary material available at 10.1186/s11689-022-09450-0.

## Background

Disruptions in sleep quality and physiology are widely prevalent and impairing in individuals with developmental neuropsychiatric disorders, including autism spectrum disorder (ASD) [1], psychotic spectrum disorders [2, 3], and attention-deficit hyperactivity disorder (ADHD) [1, 4]. Sleep has a complex, bidirectional relationship with psychiatric symptoms. For example, prolonged sleep deprivation can cause temporary perceptual distortions, disordered thinking, and hallucinations [5], while psychological distress related to psychosis worsens sleep quality and continuity. Further, a behavioral sleep intervention can improve sleep, psychosis symptoms, and psychological distress [6, 7] in patients with psychotic illness. These sleep improvements were shown to mediate changes in hallucinations and paranoia [8]. In ASD, several studies have found strong relationships between sleep disturbances and ASD-related behaviors, including restricted and repetitive behaviors, sensory sensitivities, and externalizing symptoms [9–12]. In a study of boys with ASD, sleep disturbance was found to moderate the relationship between ASD symptom severity and problem behaviors [13]. Sleep has also been shown to play a role in the executive functioning difficulties often observed in developmental psychiatric disorders [14, 15]. For example, a recent cross-sectional study found sleep mediated the relationships between real-world executive function and both overall autistic traits and restrictive and repetitive behaviors [15]. Moreover, clinical trials of effective sleep interventions in children with neurodevelopmental disorders improved a range of behavioral and mood symptoms for at least 6 months post-treatment [9–11]. Taken together, these findings offer compelling evidence that sleep may be a modifiable treatment target for psychiatric symptoms and related impairments in executive functioning, making a deep understanding of the role of sleep in these disorders an area of critical research interest. However, the lack of longitudinal studies focused on elucidating mechanistic relationships limits our current understanding.

The vast heterogeneity within psychiatric diagnostic classifications and the complex relationship between sleep, environment, genetics, and neurobiology pose challenges for studying sleep’s role in psychopathology. The gold-standard of measuring sleep, polysomnography (PSG), requires research participants to sleep in a lab, often for multiple nights, while wearing electroencephalography (EEG) and physiological sensors. This poses another challenge for sleep studies, particularly in pediatric and psychiatric populations. While parent-reported estimates of sleep (i.e., sleep duration, sleep latency) have varying validity [16–20], parent reports of overall sleep quality on sleep questions have been proven to be valid in several studies [16, 21]. Therefore, parent-report questionnaires are often the most feasible way to measure sleep; while these metrics have shown to correlate well with “gold standard” laboratory-based measures [16, 21], they cannot address the underlying biology critical for understanding sleep’s role in psychopathology. One strategy to address these challenges is to take a “genetics-first” approach of studying individuals with a known genetic etiology, such as copy number variations (CNVs) that confer large effects on psychopathology risk. Dissecting sleep changes associated with CNVs could reveal genetic influences on sleep and the brain relevant to idiopathic disorders.

The 22q11.2 locus is a genomic hotspot with highly conserved genes critical to neurodevelopment [22]. CNVs in this locus (deletions or duplications) confer some of the greatest known risks of developmental neuropsychiatric disorders [23]. 22q11.2 deletion syndrome (22qDel; also known as DiGeorge Syndrome), a 1.5 to 2.6 megabase hemizygous deletion at this locus, is one of the most common genetic syndromes, occurring in up to 1 in ~4000 live births [24]. It is associated with congenital malformations, developmental delay (DD), and intellectual disability (ID), as well as multiple psychiatric disorders including ADHD, ASD, and anxiety disorders [25]. This deletion is also one of the greatest known risk factors for developing a psychotic disorder, with up to 20-fold increased risk [26–30] compared to the general population, and an approximately 10-fold increased risk compared to populations with other neurodevelopmental disorders [31].

Notably, a duplication in the 22q11.2 region (22qDup) also confers increased risk of developmental psychiatric disorders, but occurs ~2.5 times more frequently than a deletion [23]. Relative to the 22qDel, little is known about the 22qDup phenotype. Notably, however, several recent studies reported that 22qDup is associated with a significantly lower rate of schizophrenia compared to the general population, suggesting a potential gene-dosage effect of the 22q11.2 locus on schizophrenia risk [29, 32, 33]. Further 22q11.2 gene-dosage effects have been found for both brain structure [34] and neurocognitive profiles [35]. While both the 22qDel and 22qDup confer increased risk for neurodevelopmental disorders, they differentially modulate cognitive functioning and psychosis-related symptoms [35]. Similarly, while both 22q11.2 CNVs exhibit increased ASD symptomatology compared to controls, they displayed distinct ASD symptom profiles [35].

In addition, recent evidence suggests the 22qDel confers elevated rates of sleep problems [36–39]. The proportion of 22qDel carriers with clinically significant sleep problems has been reported to range from 60 to 97% [37, 38]. Relative to controls, subjects with 22qDel report increased sleep latency, nighttime waking, sleep anxiety, sleep-disordered breathing, daytime sleepiness, and decreased sleep duration and quality [36–39]. These sleep disturbances were associated with cognitive and immune dysfunction, ADHD, and anxiety disorders [36, 37]. To our knowledge, no studies have yet investigated sleep disturbance associated with 22qDup. Despite the close associations between sleep, executive function, and psychiatric symptoms in other populations [40–42], little is known about sleep and its effect on psychiatric symptomatology in reciprocal 22q11.2 CNVs, and the current literature is limited by small sample sizes at a single timepoint. Given the high rate of sleep disturbances and neurodevelopmental disorders, and the opportunity to study gene dosage effects, investigation of reciprocal 22q11.2 CNVs offers a rich opportunity to learn about the mechanisms of sleep disturbance relevant to psychiatric disorders.

Our study aimed to address these gaps and to serve as a starting point for understanding biological underpinnings of sleep’s role in psychopathology by characterizing sleep and its effect on psychiatric symptoms across in the largest known cohort of reciprocal 22q11.2 CNV carriers. First, we aimed to examine the prevalence and severity of self-reported sleep disturbance in 22qDel and 22qDup carriers compared to controls at baseline and longitudinally over 1 year. Since the 22qDel confers an overall more severe phenotype than the 22qDup, we hypothesize that 22qDel carriers, but not 22qDup carriers, will exhibit worse sleep than controls. Due to the elevated risk of psychosis, which is associated with sleep disturbance, we also hypothesize that sleep will worsen over time in the 22qDel group but not the 22qDup group. The next aim was to test the hypothesis that sleep disturbance is an independent feature of 22q11.2 CNVs by comparing sleep disturbances between the three subject groups and controlling for medications and psychiatric comorbidities. Lastly, we sought to explore how sleep disturbances are related to common psychiatric features associated with 22q11.2 CNVs (i.e., positive psychosis-risk symptoms, ASD-related symptoms, executive function, and other emotional and behavioral problems). We expected poor sleep to have a greater effect on psychiatric symptoms in the 22qDel compared to the 22qDup group. To accomplish this, we assessed the relationship between sleep and developmental psychiatric symptoms and neurobehavioral traits cross-sectionally and longitudinally over 1 year and tested for differences in these relationships between CNV groups. Given the complex, bidirectional nature of relationships between sleep and psychiatric symptoms, we include an analysis of longitudinal data as an exploratory study of changes in sleep over time and the relationship between sleep and symptoms over time. Longitudinal assessment of sleep will inform future hypotheses for predictive analyses.

## Materials and methods

### Participants

The total sample consisted of 237 participants: 107 with a molecularly confirmed 22q11.2 deletion, 42 with a confirmed 22q11.2 duplication, and 88 healthy controls. All groups were age- and sex-matched (see Table 1 for sample demographics). Participants were a subset of those recruited for an ongoing longitudinal study conducted at University of California at Los Angeles. The majority of participants in this study were included in previous cross-sectional studies reporting on different research questions [34, 35]. For the longitudinal portion of the present study, only the first two study timepoints were included, as maximal data were available. Patients were recruited from local clinics, national support groups, and other online avenues. Control participants were recruited from the same communities as CNV carriers through online postings, local schools, pediatric clinics, and other locations in the community.

**Table 1:** Demographic and clinical characteristics at baseline and 1-year follow-up

	Typically developing control subjects	22q11.2 deletion carriers	22q11.2 duplication carriers
Baseline			
*n*_*total*_	88	107	42
*n*_*SIPS*_	74	85	34
Age, years (SD)	14.65 (7.4)	14.56 (8.0)	16.26 (13.1)
Age range, years	6–45	6–43	5–49
Males, *n* (%)	41 (47.1%)	53 (50.0%)	23 (54.8%)
Non-white, *n* (%)^a,b^	27 (30.7%)	10 (9.3%)	1 (2.4%)
Highest parental education, years (SD)^c^	16.78 (3.3)	17.17 (2.5)	15.41 (2.7)
Poor sleepers, *n* (%)^d,e^	3 (3.4%)	34 (31.8%)	14 (33.3%)
Psychosis, *n* (%)^c,d^	0 (0%)	12 (11.2%)	0 (0%)
ASD, *n* (%)^d,e^	0 (0%)	48 (44.9%)	17 (40.5%)
Medication, *n* (%)^d,e^			
Anti-psychotics	0	13 (12.1%)	4 (9.5%)
Mood stabilizers	2 (2.3%)	12 (11.4%)	3(7.1%)
Stimulants	2 (2.3%)	3 (2.9%)	6 (14.3%)
Other medication	1 (1.1%)	8 (7.6%)	2 (4.8%)
No medication	83 (94.3%)	69 (65.7%)	27 (64.3%)
One-year follow-up			
*n*_*total*_	42	62	20
*n*_*SIPS*_	32	52	17
Age, years (SD)	14.12 (5.9)	16.18 (7.2)	14.55 (9.1)
Age range, years	7–30	7–41	6–44
Males, *n* (%)	18 (43.9%)	27 (43.5%)	13 (65.0%)
Non-white, *n* (%)	8 (19.0%)	9 (14.5%)	1 (5.0%)
Highest parental education, years (SD)	17.86 (2.9)	17.00 (2.2)	15.42 (3.1)
Poor sleepers, *n* (%)^d,e^	1 (2.4%)	19 (30.6%)	7 (35.0%)
Psychosis, *n* (%)	0 (0%)	6 (9.8%)	0 (0%)
ASD, *n* (%)^d,e^	0 (0%)	22 (36.1%)	9 (45.0%)
Medication, *n* (%)^d,e^			
Anti-psychotic	1 (2.4%)	8 (13.1%)	0 (0%)
Mood stabilizer	1 (3.2%)	7 (11.5%)	6 (30.0%)
Stimulants	2 (4.9%)	3 (4.9%)	1 (5.0%)
Other medication	0 (0%)	4 (6.6%)	1 (5.0%)
No medication	37 (90.2%)	39 (63.9%)	12 (60.0%)

*ASD*, autism spectrum disorder; *n*_*SIPS*_, number of subjects included in analyses of SIPs sleep variable

^a^Control > 22qDel (*p*<0.05)

^b^Control > 22qDup (*p*<0.05)

^c^22qDel > 22qDup (*p*<0.05)

^d^22qDel > Control (*p*<0.05)

^e^22qDup > Control (*p*<0.05)

Participants with significant neurological or medical conditions (unrelated to 22q11.2 CNVs) affecting brain structure or function, previous head trauma with loss of consciousness, insufficient English fluency, and/or substance abuse/dependence within the past 6 months were excluded. All participants gave verbal and written informed consent to participate in the study. Participants under 18 years of age provided written assent and their parent/guardian provided written consent. The University of California at Los Angeles Institutional Review Board approved all study procedures and documents.

### Assessments

Clinical and cognitive assessments appropriate for participants’ developmental stage were administered to all participants (See Supplementary Table 1 for summary of age ranges for each measure). Supervised clinical psychology doctoral students administered structured psychodiagnostic evaluations to assess for psychiatric diagnoses. All diagnoses were determined by trained clinicians who participated in ongoing quality assurance and case consensus procedures. Training, reliability, and quality assurance procedures for psychodiagnostic assessments and clinician rating scales are detailed in prior publications [35, 43].

To study the relationship between sleep and clinical symptoms and neurobehavioral traits, we selected assessments measuring traits and behaviors associated with sleep in other clinical populations including ASD and clinical high-risk for psychosis populations [13, 14, 44–46]. We tested if there was an effect of sleep on positive psychosis-risk symptoms, Autism-related behaviors, real-world executive function, and emotional or behavioral problems. For a secondary analysis, if there was a significant (*q*<0.05) or marginally significant effect (*q*<0.1) of sleep on the primary outcome measure, the measure was broken down into its subscales to test for differential effects between subdomains of each trait measure (see supplementary information).

#### Psychosis-relevant measures

The Structured Interview for Psychosis-Risk Syndromes (SIPS) [47], a clinician-rated semi-structured interview, was used to measure psychosis-risk symptoms. The scale includes positive, negative, disorganized, and general symptoms and interviews both the participant and their parent to determine clinical ratings. Participants over the age of ten completed the SIPS interview, administered by a trained clinician. The primary outcome for the present analysis was positive psychosis-risk symptom score.

#### Autism spectrum measures

We chose to assess repetitive behaviors and social deficits because both of these domains are associated with sleep in studies of idiopathic ASD [9, 11, 15, 42, 48, 49]. The Repetitive Behavior Scale (RBS) [50] is a parent-report scale used to measure stereotyped, self-injurious, restrictive, need for sameness, compulsive, and ritualistic behaviors. A higher score on the RBS subscales indicates an increased frequency of behaviors. To measure social communication deficits, we used the Social Responsiveness Scale-2^nd^ edition (SRS) [51], a parent-report rating scale measuring dysfunctional social behavior with the subdomains: awareness, cognition, communication, autistic traits, and motivation. Further information on scoring methods is detailed in Jalal et al. [52].

#### Real-world executive function

The Behavior Rating Inventory of Executive Function [53] (BRIEF) was used to measure parent-reported real-world executive function in participants aged 6–18. A previous study in a population-based cohort found associations between sleep, autistic traits, and the BRIEF [15], making it a measure of interest for the present study. The BRIEF consists of an overall global composite score and eight clinical subscales including shifting, emotional, monitoring, inhibition, plan/organize, and organization/material. The overall global composite score was used as the primary outcome of real-world executive function.

#### Emotional or behavioral problems

We also investigated the relationship between sleep and emotional or behavioral problems on the Child Behavior Checklist (CBCL) [54, 55]. The CBCL subdomains are anxious/depressed, withdrawn/depressed, somatic complaints, social problems, attention problems, rule breaking, aggressive behavior, and thought problems. The CBCL was completed by the parents of participants aged 6–18.

#### Sleep measure

To measure sleep disturbance on a continuous scale, we used item G1 from the SIPS (SIPSG1), which rates level of sleep disturbance on a six-point scale. A rating of six corresponds with severe sleep disturbance. The SIPS has not been validated in children under the age of 10, therefore analyses using this variable did not include participants younger than 10. To include the entire age range of our cohort in analyses of sleep effects on psychiatric symptoms, we created a categorical variable which classified participants as either good or poor sleepers. Previous studies in children with developmental psychiatric disorders have validated the use of a categorical variable based on parent-reported sleep using sleep questionnaires, actigraphy, and PSG [42, 56]. Participants 10 years and older were classified as poor sleepers if they scored a 3 or higher on SIPSG1. Participants 10 years or younger were classified as a poor sleeper if they scored 2 on the CBCL checklist item 100 (CBCL100). CBCL100 asked parents to rate their child’s trouble sleeping on a scale from 0 to 2 (not true to very true or often true). The comparability of this categorical sleep variable was verified through correlations between the CBCL100 and SIPSG1 (see Supplementary Figure 1, Supplementary Tables 1, 2) and sensitivity analyses which exclude subjects who did not complete the SIPS (see Supplementary Figures 2, 3 and Supplementary Tables 3, 4). CBCL100 and SIPSG1 were removed from total scores on the SIPS and CBCL. CBCL100 has been validated to be concordant with sleep questionnaires, sleep diaries, actigraphy, and PSG [57, 58] while SIPSG1 has been used to measure sleep disturbance in numerous studies of individuals at high-risk for developing a psychotic disorder [59–61]

### Statistical analyses

All statistical analyses were performed, and figures created, in R 4.0.2 [62] using the packages lme4 [63] and ggplot2 [64]. To address the question of how sleep disturbance in reciprocal 22q11.2 CNV carriers compares to controls, we conducted linear mixed models with sleep as the dependent variable (using the continuous variable) and participant group (22qDel vs. 22qDup vs. control, with control as the reference group) as the independent variable. We analyzed each model cross-sectionally (at baseline) and longitudinally (two timepoints across approximately 1 year). The longitudinal model included a group-by-time interaction. All models included age and biological sex as covariates and participant ID as the random effects term. To test for differences between CNV groups, we re-ran the same models including only participants in the CNV groups (22qDel as the reference group). To determine if differences in sleep between CNV groups and controls could be attributed to differences in psychiatric comorbidities or medications between groups, we independently added variables measuring attention, current medication, Full Scale IQ, ASD diagnosis, and psychotic disorder diagnosis into the models to test if the group difference remained statistically significant when accounting for these variables. Within each model, a false discovery rate (FDR) correction was applied on the effects of subject group.

To test the relationship between sleep disturbance and clinical symptoms, we conducted linear mixed models with each symptom domain as the dependent variable and the sleep categorization (good vs. poor sleeper) and participant group (22qDel vs. 22qDup; 22qDel as the reference group) as independent variables. Cross-sectional models included a group-by-sleep interaction and longitudinal models included a group-by-time interaction. All models included age and biological sex as covariates and participant ID as the random effects term. Due to small number of poor sleepers in the control group, only CNV carriers were included in analyses of psychiatric symptoms. To control for multiple comparisons within each domain, FDR correction was applied. Only the main effects of sleep, group, and group-by-sleep interaction were included in the FDR correction, since effects of time, age, and sex are not the primary variables of interest in this study. A *q*-value <0.05 was considered statistically significant.

## Results

### Participant demographics

Demographic variables for each group at each timepoint are reported in Table 1. As expected, both 22qDel and 22qDup groups showed significantly elevated rates of ASD diagnoses and medication use at both timepoints compared to controls. Consistent with previous literature [32], 22qDel reported significantly higher rates of psychotic disorders compared to the other two groups. There was also a significant difference in highest parental education level between the 22qDel and 22qDup group. There were no significant differences in demographic or clinical characteristics of the samples between baseline and 1-year follow-up (*p* >0.196).

### Comparison of sleep disturbance between 22q11.2 CNV carriers and controls

**Fig. 1:**
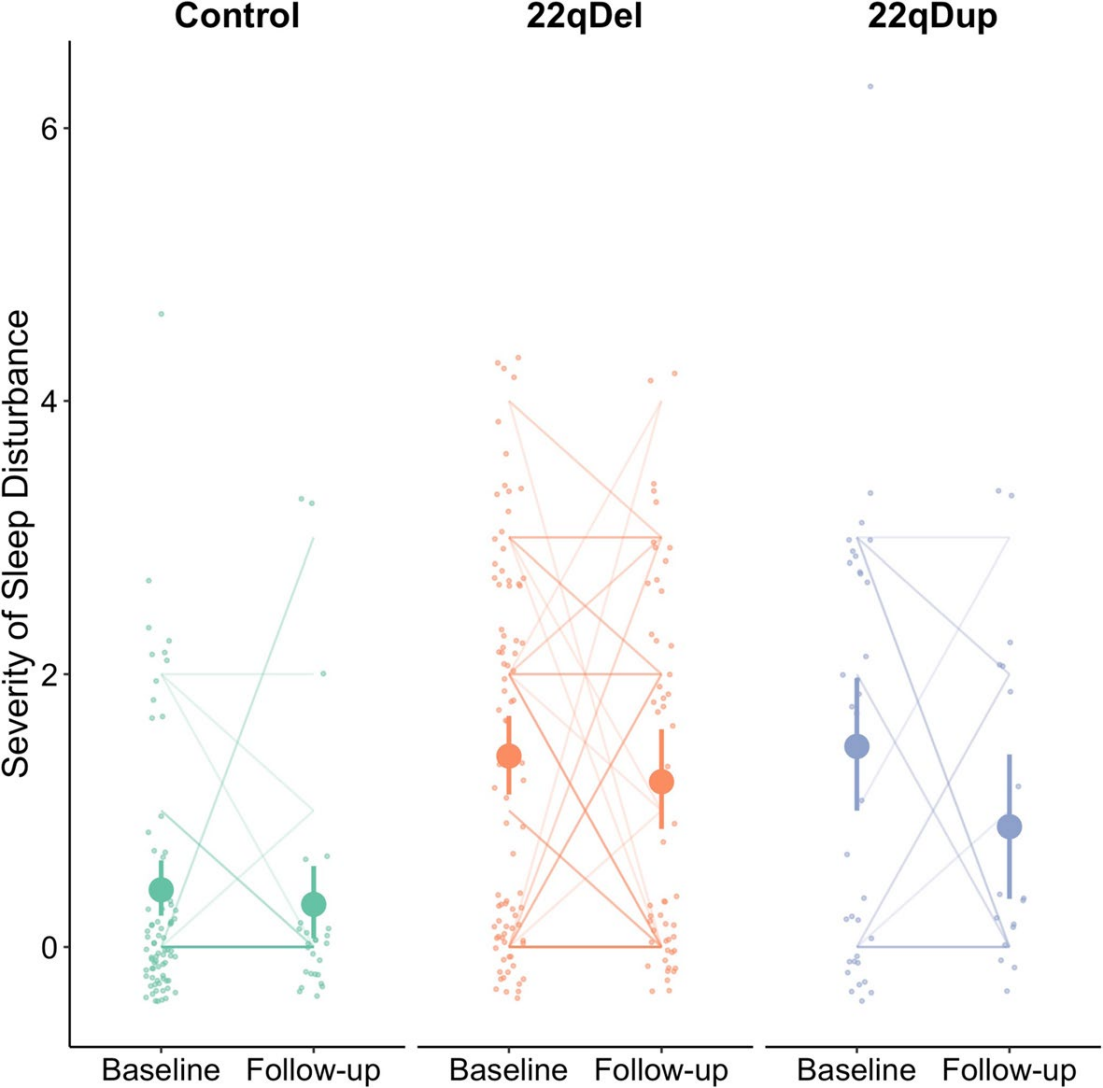
Quantitative measure of sleep disturbance across groups. Sleep disturbance, assessed via the SIPS, is significantly worse in CNV carriers compared to controls cross-sectionally (*q*<0.001) and longitudinally (*q*<0.001). However, there is no significant difference in sleep quality between CNV groups (*q*>0.940). Large dots represent the group mean and 95% confidence interval at each timepoint

Both 22q11.2 deletion and duplication groups reported significantly greater sleep disturbance on the SIPS compared to controls, cross-sectionally and longitudinally (Fig. 1, Table 2). Sleep disturbance severity was not statistically different between 22qDel and 22qDup in either model (cross-sectional: *b*=0.015, *q*=0.940; longitudinal: *b*=0.006, *q=*0.982). Sleep disturbance did not change over time (*b*=−0.074, *p=*0.754), and there were no statistically significant group-by-time interactions. There was a significant effect of age both cross-sectionally and longitudinally such that older subjects slept worse than younger subjects (cross-sectional: *b=0.198*, *p*=0.004; longitudinal: *b*=0.213, *p*<0.001). These results are summarized in Table 2. About one-third of both CNV groups at each timepoint met the poor sleeper criteria, while <4% of controls met these criteria (Table 1).

**Table 2:** Differences in SIPS-measured sleep disturbance between groups

Covariates	Effect of time		Effect of covariate			Effect of subject group			Group × time interaction	
Cross-sectional	*β*	*p-value*	*Group*	*β*	*p-value*	*Group*	*β*	*q-value*	*β*	*p-value*
Age + sex	-	-	-	**0.198**	**0.004**	Del	**0.728**	**<0.001**	-	-
			**-**	**-**	**-**	Dup	**0.741**	**<0.001**	-	-
Age+ sex + attention	-	-	**-**	**0.222**	**0.015**	Del	**0.525**	**0.009**	-	-
			**-**			Dup	**0.592**	**0.014**	-	-
Age+ sex + medication	-	-	Antipsychotic	**0.557**	**0.026**	Del	**0.580**	**<0.001**	-	-
			Mood Stabilizer	0.242	0.354	Dup	**0.675**	**<0.001**	-	-
			Stimulant	−0.027	0.932	-	-	-	-	-
			Other	0.521	0.107	-	-	-	-	-
Age + sex + IQ	-	-	-	−0.128	0.244	Del	**0.516**	**0.026**	-	-
						Dup	0.500	0.070	-	-
Age + sex + ASD	-	-	-	0.033	0.643	Del	**0.782**	**<0.001**	-	-
						Dup	**0.811**	**0.001**	-	-
Age + sex + PSY	-	-	-	0.558	0.054	Del	**0.648**	**<0.001**	-	-
						Dup	**0.743**	**<0.001**	-	-
**Longitudinal**	*β*	*p-value*	*Group*	*β*	*p-value*	*Group*	*β*	*q-value*	*β*	*p-value*
Age + sex	−0.074	0.754		**0.213**	**<0.001**	Del	**0.747**	**<0.001**	−0.122	0.601
						Dup	**0.747**	**<0.001**	−0.325	0.289
Age+ sex + attention	−0.214	0.276	**-**	**0.222**	**0.003**	Del	**0.546**	**0.005**	0.023	0.927
						Dup	**0.611**	**0.010**	−0.273	0.421
Age+ sex + medication	−0.074	0.690	Antipsychotic	**0.537**	**0.013**	Del	**0.600**	**<0.001**	−0.106	0.654
			Mood Stabilizer	0.321	0.109	-	-	-	-	-
			Stimulant	0.110	0.657	-	-	-	-	-
			Other	0.430	0.099	Dup	**0.662**	**<0.001**	−0.363	0.261
Age + sex + IQ^a^	−0.196	0.312	-	−0.102	0.299	Del	**0.574**	**0.011**	−0.130	0.595
						Dup	0.522	0.056	-	-
Age + sex + ASD	−0.061	0.740	-	−0.048	0.387	Del	**0.669**	**<0.001**	−0.085	0.725
						Dup	**0.647**	**0.005**	−0.312	0.314
Age + sex + PSY	−0.055	0.767	-	0.305	0.118	Del	**0.703**	**<0.001**	−0.120	0.615
						Dup	**0.748**	**<0.001**	−0.381	0.229

^a^Insufficient longitudinal IQ data in 22q11.2 duplication group

As shown in Table 2, the differences in the continuous sleep measure between CNV groups and controls remained significant when medication, attention, ASD diagnosis, and psychotic disorder diagnosis were each added to the models. Both cross-sectionally and longitudinally, subjects on antipsychotics reported greater sleep disturbance compared to those on no medication. Across groups at each timepoint, there was a main effect of attention on sleep such that individuals with more attention problems reported worse sleep disturbance. The effect of ASD and the effect of psychotic disorders on sleep disturbance did not reach significance. There were no significant differences in sleep between CNV groups when medication, attention, ASD diagnosis, and psychotic disorder diagnosis were included in the models.

When IQ was added to both models, the difference in sleep between 22qDel and controls remained significant, but the difference between 22qDup and controls was attenuated to a trend level (Table 2). There remained no significant differences in sleep disturbance severity between CNV groups when IQ was added to the model.

### Effect of sleep on positive psychosis-risk and autism-related symptoms

**Fig. 2:**
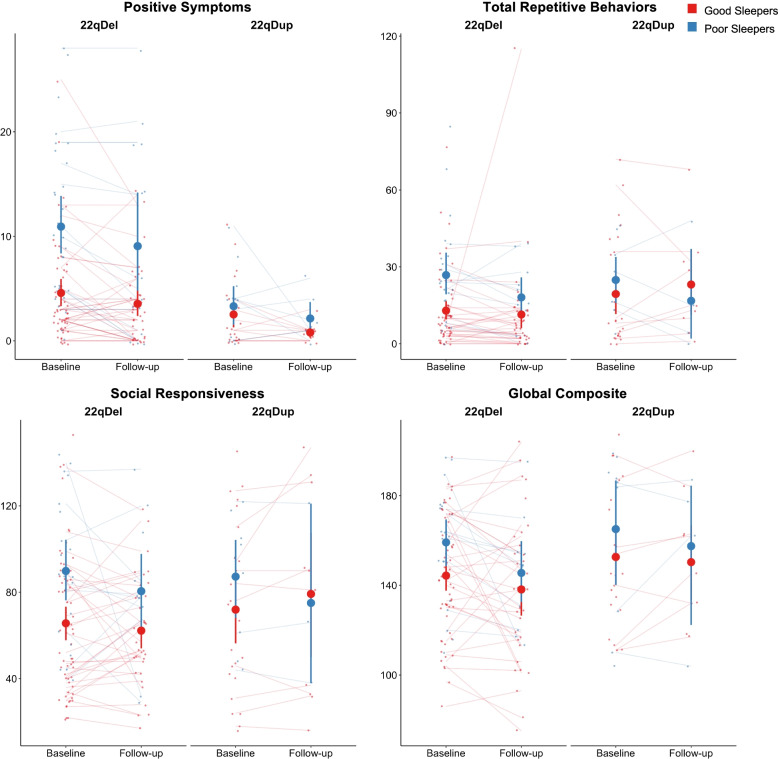
Clinical measures in good and poor sleepers at each timepoint within 22q11.2 CNV groups. In both cross-sectional and longitudinal analyses across CNV groups, poor sleepers score higher (i.e., more pathological) on positive symptoms (cross-sectional: *q*=<0.001, longitudinal: *q*<0.001), total repetitive behaviors (cross-sectional: *q*= 0.005, longitudinal: *q*=0.018), and social responsiveness (cross-sectional: *q*= 0.026, longitudinal: *q*=0.015 ). The effect of sleep on BRIEF global composite score failed to survive FDR correction (cross-sectional: *p*= 0.0, longitudinal: *p*=0.0). Large dots represent the group mean and 95% confidence interval for good and poor sleepers at each timepoint

**Fig. 3:**
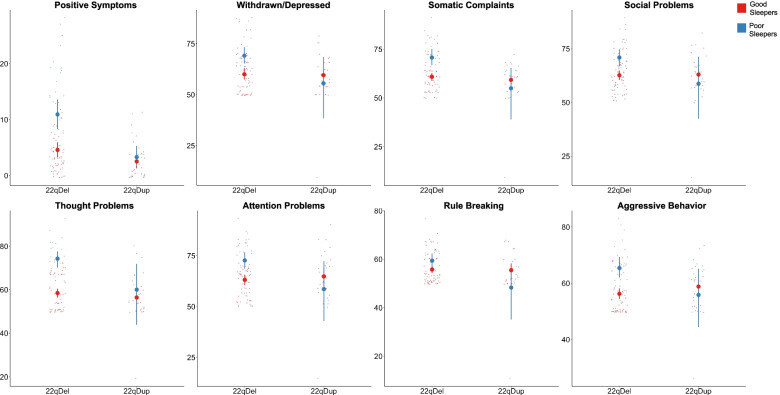
Baseline CNV group-by-sleep interactions. There were significant group-by-sleep interactions at baseline for positive psychosis-risk symptoms (*q=*0.041), and CBCL-measured withdrawn/depressed (*q=*0.020), somatic complaints (*q=*0.007), social problems (*q=*0.017), thought problems (*q=*0.012), attention problems (*q=*0.009), rule-breaking (*q=*0.006), and aggressive behaviors subscales (*q=*0.004). Large dots represent the group mean and 95% confidence interval for good and poor sleepers in each subject group

Across CNV groups, poor sleepers reported more severe positive psychosis-risk symptoms than good sleepers, both cross-sectionally (Table 3) and longitudinally (Table 4), as shown in Fig. 2. There was a significant group-by-sleep interaction for positive symptoms at baseline (Fig. 3). There was a bigger difference in positive symptoms between good and poor sleepers in the 22qDel group compared to the 22qDup group. 22qDel subjects reported higher severity of positive psychosis-risk symptoms longitudinally compared to 22qDup.

**Table 3:** Results of cross-sectional models

	Effect of sleep		Effect of CNV group		Group×sleep interaction	
	*β*	*q-value*	*β*	*q-value*	*β*	*q-value*
Positive symptoms	**0.976**	**<0.001**	−0.348	0.180	**−0.855**	**0.041**
Total repetitive behaviors	**0.787**	**0.005**	0.357	0.201	−0.494	0.275
Social responsiveness	**0.652**	**0.026**	0.247	0.438	−0.238	0.595
BRIEF global composite	0.559	0.087	0.261	0.444	−0.115	0.804
Anxious/depressed	**0.876**	**0.003**	−0.086	0.927	−0.836	0.083
Withdrawn/depressed	**0.738**	**0.007**	−0.022	0.927	**−1.143**	**0.020**
Somatic complaints	**0.943**	**<0.001**	−0.149	0.927	**−1.370**	**0.007**
Social problems	**0.737**	**0.007**	0.048	0.927	**−1.183**	**0.017**
Thought problems	**1.420**	**<0.001**	−0.184	0.927	**−1.105**	**0.012**
Attention problems	**0.795**	**0.006**	0.262	0.927	**−1.342**	**0.009**
Rule breaking	**0.617**	**0.017**	−0.076	0.927	**−1.438**	**0.006**
Aggressive behavior	**1.203**	**<0.001**	0.261	0.927	**−1.483**	**0.004**

**Table 4:** Results of longitudinal models

	Effect of sleep		Effect of CNV group		Effect of time		Group×time interaction	
	*β*	*q-value*	*β*	*q-value*	*β*	*p-value*	*β*	*p-value*
Positive symptoms	**0.706**	**<0.001**	**−0.675**	**<0.001**	**−0.207**	**0.005**	0.044	0.750
Total repetitive behaviors	**0.521**	**0.018**	0.213	0.307	−0.055	0.694	−0.015	0.957
Social responsiveness	**0.546**	**0.015**	0.166	0.418	−0.136	0.216	0.263	0.232
BRIEF global composite	0.425	0.065	0.179	0.394	**−0.255**	**0.042**	0.358	0.155
Anxious/depressed	**0.619**	**0.005**	−0.305	0.237	−0.200	0.247	0.067	0.840
Withdrawn/depressed	0.355	0.082	−0.328	0.237	**−0.348**	**0.049**	0.203	0.543
Somatic complaints	**0.461**	**0.038**	−0.511	0.064	−0.266	0.129	**0.664**	**0.047**
Social problems	0.362	0.081	−0.270	0.282	−0.194	0.314	−0.020	0.958
Thought problems	**1.038**	**<0.001**	−0.490	0.064	0.049	0.763	0.135	0.661
Attention problems	0.421	0.061	−0.202	0.401	−0.253	0.149	0.194	0.560
Rule breaking	0.247	0.195	−0.448	0.101	0.080	0.675	0.009	0.980
Aggressive behavior	**0.677**	**0.002**	−0.100	0.624	−0.011	0.947	0.010	0.974

There was a significant effect of sleep categorization on total RBS and total SRS scores at baseline (Table 3, Fig. 2) and across timepoints (Table 4; Supplementary Figure 2). There were no significant group-by-sleep interactions for any autism-related domains.

### Effect of sleep on real-world executive function

Poor sleepers, across CNV groups, scored worse on the global composite overall index on the BRIEF, both cross-sectionally (Table 3, Fig. 2) and longitudinally (Table 4, Fig. 2), although the effect of sleep in both models was marginally significant.

### Effect of sleep on behavioral or emotional problems

**Fig. 4:**
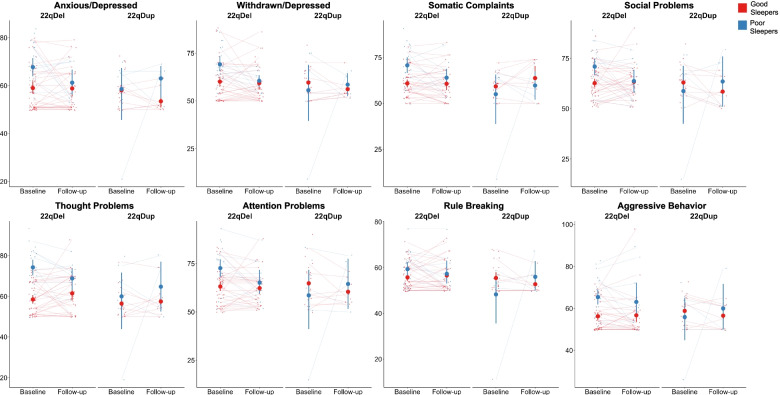
CBCL subdomain scores in good and poor sleepers at each timepoint within 22q11.2 CNV groups. At baseline, there was a significant main effect of sleep category on all CBCL subdomains (*q*<0.017). Across timepoints, there was a significant main effect of sleep category on anxious/depressed (*q*=0.005), somatic complaints (*q*=0.038), thought problems (*q*<0.001), and aggressive behavior (*q*=0.002) subscales. There was a trend towards a significant effect of sleep category on attention problems (*q*=0.061). Large dots represent the group mean and 95% confidence interval for good and poor sleepers at each timepoint

Cross-sectionally across CNV groups, poor sleepers scored significantly higher on all subdomains of the CBCL (Table 3, Fig. 4). There were significant group-by-sleep interactions on all subdomains except anxious/depressed, such that there was a larger difference between good and poor sleepers in 22qDel carriers compared to 22qDup carriers (Fig. 3). Longitudinally, poor sleepers across CNV groups scored significantly higher on the anxious/depressed, somatic complaints, thought problems, and aggressive behaviors subdomains. Poor sleepers scored marginally higher than good sleepers on the attention subscale. There was a trend towards a main effect of group, in which the 22qDel group scored higher than the 22qDup group on somatic complaints and thought problems.

## Discussion

In this study, we report four core, novel findings related to sleep and psychiatric symptomatology in 22q11.2 CNV carriers. First, similar to 22qDel, 22qDup was associated with more severe sleep disturbances than typically developing controls. Second, we found these sleep disturbances were independent of psychiatric conditions and medication usage. Third, sleep difficulties negatively impacted psychiatric symptomatology in both 22qDel and 22qDup carriers. Lastly, sleep disturbance had a differential impact on particular psychiatric symptoms in 22q11.2 CNV carriers; specifically, positive psychosis-risk symptoms, withdrawn/depressed symptoms, somatic complaints, social problems, thought problems, attention problems, rule breaking, and aggressive behavior.

In our first and second aims, we found both 22q11.2 CNV groups reported significantly poorer sleep than controls, cross-sectionally and longitudinally, even when controlling for psychiatric comorbidities and medications, supporting our hypothesis that sleep disturbance is an independent feature of 22q11.2 CNVs. Contrary to expectations, there was no difference in reported sleep disturbance between the two CNV groups. Increased sleep difficulty in 22qDel has been reported in previous cross-sectional studies [36–39]. In our study, about one-third of subjects in both the 22qDel and 22qDup groups were classified as poor sleepers, while prevalence rates of clinically significant sleep problems was previously reported to be greater than 60% in 22qDel carriers [37, 38]. This discrepancy could be attributed to different questionnaires used to classify significant sleep problems, and differing age groups. Our study reports on a cohort with a wide age range, which includes adult participants, while previous studies focused exclusively on children. This is the first study to investigate sleep disturbance in 22qDup carriers, so there is no data to compare with our cohort.

In the last aim, we found that across CNV groups, poor sleepers reported elevated psychiatric symptomatology relative to good sleepers. Poor sleep had a consistently strong effect across psychosis-risk symptoms subscales (SIPS) and emotional and behavior problems reported on the CBCL, similar to findings observed in idiopathic psychiatric disorders [59, 65]. This finding is also consistent with and builds upon prior literature in 22qDel carriers, which found poor sleep is associated with anxiety disorders, ADHD, and conduct disorders [37]. Poor sleep also had a strong effect on ASD-related measures (RBS, SRS) with poor sleepers exhibiting increased severity of repetitive behaviors and social impairments. This finding is consistent with a previous study in idiopathic ASD that found that sleep fragmentation was associated with an increased RBS total score and poor sleepers scored higher on ritualistic and compulsive subscales of the RBS compared to good sleepers with ASD [56]. Sleep fragmentation was associated with RBS total score [56]. Also consistent with previous findings in idiopathic ASD [46], we found total SRS score was significantly associated with sleep cross-sectionally and longitudinally, with poorer sleep being associated with elevated SRS scores over time. Contrary to expectations, the effect of sleep on the BRIEF global composite did not survive FDR correction at *q<0.05* in either model. However, there was a trend towards a significant effect of sleep on the global composite score such that poor sleepers performed worse across CNV groups in both models. This trend is consistent with a previous study reporting sleep deficits were associated with a worse global composite score on the BRIEF [15]. Theoretically, it is possible that the BRIEF measures are not impacted by acute poor sleep as much as the other clinical measures. Perhaps chronic poor sleep over an extended period of time has a stronger impact on executive function than acute sleep disturbance. The data assessed in this study was not able to address this possibility, since we did not collect information on sleep disturbance duration.

We found sleep has a greater effect on psychiatric symptomatology—namely, positive psychosis-risk symptoms, and withdrawn/depressed, somatic complaints, social problems, thought problems, attention problems, rule breaking, and aggressive behaviors—in 22qDel carriers relative to 22qDup carriers. This suggests 22qDel carriers are more susceptible to the effects of poor sleep compared to 22qDup, which is consistent with the fact that the 22qDel often confers a more severe phenotype compared to the 22qDup [23]. This finding is also consistent with recent cross-sectional findings from our group that the 22qDel has a greater effect on IQ, psychosis-risk symptoms, and brain morphometry compared to the 22qDup [34, 35]. The differential impact of sleep on psychiatric symptoms in 22q11.2 CNV carriers suggests that gene dosage at the 22q11.2 locus may influence mechanisms involved in sleep’s relationship with symptoms of developmental psychiatric disorders. Further, it provides evidence for a genetic-basis of sleep disturbance in 22q11.2 CNVs, and perhaps idiopathic disorders, such as schizophrenia and ASD. This conclusion is supported by a recent study of the 22q11.2 locus in *drosophila*, which found knockout of the LZTR1 homolog gene on the 22q11.2 locus caused widespread sleep disturbance [66]. Further experiments revealed this gene affects sleep through GABAergic signaling, disruptions in which are also implicated in schizophrenia and ASD [67–71]. Taken together, these results suggest a genetic basis for sleep disturbance in 22q11.2 CNV carriers and associated psychiatric disorders. Further studies are required, in both humans and animal models, in order to identify underlying genetic and neurobiological mechanisms of sleep in 22q11.2 CNV carriers and determine how 22q11.2 genes impact the relationship between sleep and psychiatric symptoms, and if genes within this locus could contribute to sleep’s role in idiopathic disorders. Future studies should include measures of brain circuitry, sleep physiology, and gene expression.

In addition to studying the association between sleep and 22q11.2 CNVs, we also report a number of group differences between 22qDel and 22qDup carriers that have not previously been reported. First, as in Lin et al. [35], we found decreased stereotyped behaviors and increased positive and negative psychosis-risk symptoms in 22qDel carriers compared to 22qDup carriers. Expanding upon this finding, we also found 22qDel carriers reported more severe disorganized—but not general—psychosis-risk symptoms compared to 22qDup. On the CBCL, there was a trend towards a significant group difference in thought problems and somatic complaints. 22qDel carriers reported more thought problems than 22qDup carriers, which is consistent with increased rates of psychotic disorders in 22qDel carriers and decreased rates in 22qDup carriers [26–30, 32, 33]. Additionally, 22qDel carriers reported more somatic complaints, which could be attributed to increased medical problems reported in this population.

Generally, sleep disturbance is most common in adolescence and older age [72]. So, it is unsurprising that sleep disturbance worsened with older age in our analysis. However, a thorough investigation of sleep across the lifespan would likely require advanced methods of measuring sleep and analytical methods appropriate for testing non-linear effects, which is outside the scope of the present study. Additionally, there was no significant change is sleep disturbances across time in our sample. This could be due to a number of factors. First, since there was an effect of age, it is possible that the 1-year duration between visits was too short to observe changes in sleep disturbance. For example, the increased sleep disturbance in adolescence implies that one’s sleep would worsen more between ages 10 and 15 compared to between ages 10 and 11. So, while our study is the first investigation of longitudinal changes in sleep in 22q11.2 CNV carriers, it is possible that our data is not properly equipped to explore changes in sleep over time in this population. To address this question and to fully understand age-related changes in sleep, large-scale longitudinal assessments employing objective measures of sleep across several years are required.

In addition to contributing to the mechanistic understanding of the relationship between sleep and psychopathology, our findings also inform clinical knowledge of treating psychiatric symptoms in 22q11.2 CNV carriers. Our study suggests that an intervention that directly targets sleep could have multiple beneficial effects, with downstream influences on psychiatric symptoms and behavioral problems across diagnostic classifications in 22q11.2 CNV carriers. Further research is needed to test the effectiveness of individual sleep treatments in this population. Potential lines of study include clinical trials in 22q11.2 CNV carriers of behavioral sleep therapies, which have been effective in psychotic populations [6, 73], and melatonin, which has been effective in ASD populations [9, 11]. These clinical trials should not only test the ability of an intervention to treat sleep disturbance, but also its impact on neuropsychiatric symptoms.

As with most studies of rare disorders, there are several limitations of this study to consider. We were unable to collect objective, laboratory-based measures of sleep and did not administer a comprehensive sleep questionnaire. For younger participants, sleep disturbance scores were collected solely via parent report, while older subjects underwent a clinical interview. While we acknowledge the limitations of this measure, we found our two sleep measures were highly correlated. Further, a recent study found that parent-reported subjective sleep report was as valid as participant-reported subjective sleep report when compared to PSG recording [20] and another study concluded parental report of overall sleep quality and symptoms, similar to the methods used in the current study, is reliable [16]. However, it is important to consider that subjective sleep report and PSG-derived sleep measures capture two related but distinct sleep-related processes. While subjective sleep measures are valid, they do not capture information about sleep physiology and neurobiology, meaning we cannot make inferences about biological underpinnings of the observed relationships. Thus, our study focuses only on subjectively reported sleep disturbance and does not make inferences about objective measures of sleep or sleep physiology. Further, the nature of our sleep variable does not indicate the type of sleep disturbance exhibited, only its presence and severity. 22q11.2 CNVs are associated with craniofacial abnormalities, which can lead to increased rates of sleep-disordered breathing (SDB), such as obstructive sleep apnea [74, 75]. Sleep disturbance related to SDB confers a different clinical phenotype and affects downstream mechanisms differently than a sleep disturbance related to insufficient sleep [76, 77]. While it is possible that the observed difference in sleep disturbance between 22q11.2 CNV carriers and controls could be driven by higher rates of SDB in 22q11.2 CNV carriers, rates of airway problems and/or craniofacial abnormalities did not differ between poor sleepers and good sleepers (see Supplementary Information), so it is unlikely that these medical conditions accounted for the increased rates of poor sleepers. Nevertheless, future investigation of 22q11.2 CNV carriers should consider the differential impacts of sleep breathing disorders and disorders of insufficient sleep on brain structure, psychiatric symptoms, and neurobehavioral traits.

Another limitation to consider is the small sample size of the 22qDup group compared to that of the 22qDel and control groups. Unlike 22qDel carriers, 22qDup carriers often are not identified through clinical genetic testing due to the highly variable phenotype and less frequent medical comorbidity [23] , posing a recruitment challenge for this population. While the cohort of 22qDup included here is relatively modest, it is one of the largest cohorts reported in the literature to date. The challenge of recruiting subjects with a rare disorder (22qDel carriers and 22qDup carriers) led to a sample that is not racially diverse, an issue that we are attempting to better address in new studies underway. As a result, there were significantly fewer non-white participants in the 22qDel and 22qDup groups compared to the control group. Although unlikely, it is possible that this difference impacted the comparison of sleep across the three study groups.

## Conclusions

In summary, our findings establish that the 22q11.2 locus impacts sleep which, in turn, associates with psychopathology. Further, sleep disturbances can differentially affect psychopathology, depending on gene dosage. These results contribute to our understanding of psychiatric symptoms in 22q11.2 CNV carriers and offer a potential intervention target. Our findings serve as a starting point for exploring a genetic basis for sleep disturbance in neurodevelopmental psychiatric disorders.

## Supplementary Information

**Additional file 1: Supplementary Table 1.** Summary of clinical measures, validated age range, age range of sample, reporter, and number of subjects who completed the measure. **Supplementary Table 2.** Scoring of Sleep Disturbance Item on SIPS. **Supplementary Figure 1.** Correlation between sleep items on SIPS and CBCL. **Supplementary Table 3.** Categorization of Participants According to Each Sleep Measure. **Supplementary Figure 2.** Summary scores of 22q11.2 CNV carriers who completed SIPS. **Supplementary Figure 3.** CBCL Scores of 22q11.2 CNV carriers who completed SIPS. **Supplementary Table 4.** Results of cross-sectional model including only participants who completed the SIPS sleep measure. **Supplementary Table 5.** Results of longitudinal models including only participants who completed SIPS sleep measure. **Supplementary Table 6.** Results of cross-sectional models. **Supplementary Table 7.** Results of longitudinal models. **Supplementary Figure 4.** SIPS subdomain scores in good and poor sleepers at each timepoint within 22q11.2 CNV groups. **Supplementary Figure 5.** RBS subdomain scores in good and poor sleepers at each timepoint within 22q11.2 CNV groups. **Supplementary Figure 6.** SRS subdomain scores in good and poor sleepers at each timepoint within 22q11.2 CNV groups. **Supplementary Figure 7.** BRIEF subdomain scores in good and poor sleepers at each timepoint within 22q11.2 CNV groups.

## Data Availability

The datasets used and/or analyzed during the current study are available from the corresponding author on reasonable request.

## Abbreviations

22qDel: 22q11.2 deletion
22qDup: 22q11.2 duplication
ADHD: Attention-deficit hyperactivity disorder
ASD: Autism spectrum disorder
BRIEF: Behavior Rating Inventory of Executive Function
CBCL: Child Behavior Checklist
CBCL100: Child Behavior Checklist item 100
CNV: Copy number variation
DD: Developmental delay
EEG: Electroencephalography
FDR: False discovery rate
ID: Intellectual disability
PSG: Polysomnography
RBS: Repetitive Behavior Scale
SIPS: Structured Interview for Psychosis-Risk Symptoms
SIPSG1: Structured Interview for Psychosis-Risk Syndromes item G1
SRS: Social Responsiveness Scale

## References

[1] Singh K, Zimmerman AW (2015). Sleep in autism spectrum disorder and attention deficit hyperactivity disorder. Semin Pediatr Neurol.

[2] Hertenstein E, Feige B, Gmeiner T, Kienzler C, Spiegelhalder K, Johann A (2019). Insomnia as a predictor of mental disorders: a systematic review and meta-analysis. Sleep Med Rev.

[3] Mollon J, Knowles EEM, Mathias SR, Gur R, Peralta JM, Weiner DJ, et al. Genetic influence on cognitive development between childhood and adulthood. Mol Psychiatry. 2018. 10.1038/s41380-018-0277-0 Springer US. 10.1038/s41380-018-0277-0 PMC6570578 30644433

[4] Hvolby A (2015). Associations of sleep disturbance with ADHD: implications for treatment. ADHD Atten Deficit Hyperact Disord.

[5] Waters F, Chiu V, Atkinson A, Blom JD. Severe sleep deprivation causes hallucinations and a gradual progression toward psychosis with increasing time awake. Front Psychiatry. 2018;9:303. 10.3389/fpsyt.2018.00303 PMC6048360 30042701

[6] Chiu VW, Ree M, Janca A, Iyyalol R, Dragovic M, Waters F (2018). Sleep profiles and CBT-I response in schizophrenia and related psychoses. Psychiatry Res.

[7] Myers E, Startup H, Freeman D (2011). Cognitive behavioural treatment of insomnia in individuals with persistent persecutory delusions: a pilot trial. J Behav Ther Exp Psychiatry.

[8] Freeman D, Sheaves B, Goodwin GM, Yu LM, Nickless A, Harrison PJ (2017). The effects of improving sleep on mental health (OASIS): a randomised controlled trial with mediation analysis. Lancet Psychiatry.

[9] Yuge K, Nagamitsu S, Ishikawa Y, Hamada I, Takahashi H, Sugioka H (2020). Long-term melatonin treatment for the sleep problems and aberrant behaviors of children with neurodevelopmental disorders. BMC Psychiatry.

[10] Papadopoulos N, Sciberras E, Hiscock H, Mulraney M, McGillivray J, Rinehart N (2019). The efficacy of a brief behavioral sleep intervention in school-aged children with ADHD and comorbid autism spectrum disorder. J Atten Disord.

[11] Malow B, Adkins KW, McGrew SG, Wang L, Goldman SE, Fawkes D (2012). Melatonin for sleep in children with autism: a controlled trial examining dose, tolerability, and outcomes. J Autism Dev Disord.

[12] Schroder CM, Malow BA, Maras A, Melmed RD, Findling RL, Breddy J (2019). Pediatric prolonged-release melatonin for sleep in children with autism spectrum disorder: impact on child behavior and caregiver’s quality of life. J Autism Dev Disord.

[13] Lindor E, Sivaratnam C, May T, Stefanac N, Howells K, Rinehart N (2019). Problem behavior in autism spectrum disorder: considering core symptom severity and accompanying sleep disturbance. Front Psychiatry.

[14] Elkhatib Smidt SD, Ghorai A, Taylor SC, Gehringer BN, Dow HC, Langer A (2022). The relationship between autism spectrum and sleep–wake traits. Autism Res.

[15] Tsai T-H, Chen Y-L, Gau SS-F. Relationships between autistic traits, insufficient sleep, and real-world executive functions in children: a mediation analysis of a national epidemiological survey. Psychol Med. 2019;51:579–86. 10.1017/S0033291719003271 31769374

[16] Dayyat EA, Spruyt K, Molfese DL, Gozal D (2011). Sleep estimates in children: parental versus actigraphic assessments. Nat Sci Sleep.

[17] Li L, Sheehan CM, Valiente C, Eisenberg N, Doane LD, Spinrad TL (2021). Similarities and differences between actigraphy and parent-reported sleep in a Hispanic and non-Hispanic White sample. Sleep Med.

[18] Mazza S, Bastuji H, Rey AE (2020). Objective and subjective assessments of sleep in children: comparison of actigraphy, sleep diary completed by children and parents’ estimation. Front psychiatry.

[19] Holzhausen EA, Hagen EW, LeCaire T, Cadmus-Bertram L, Malecki KC, Peppard PE (2021). A comparison of self- and proxy-reported subjective sleep durations with objective actigraphy measurements in a survey of Wisconsin children 6-17 years of age. Am J Epidemiol.

[20] Combs D, Goodwin JL, Quan SF, Morgan WJ, Hsu CH, Edgin JO (2019). Mother knows best? Comparing child report and parent report of sleep parameters with polysomnography. J Clin Sleep Med.

[21] Iwasaki M, Iwata S, Iemura A, Yamashita N, Tomino Y, Anme T (2010). Utility of subjective sleep assessment tools for healthy preschool children: a comparative study between sleep logs, questionnaires, and actigraphy. J Epidemiol.

[22] Guna A, Butcher NJ, Bassett AS. Comparative mapping of the 22q11.2 deletion region and the potential of simple model organisms. J Neurodev Disord. 2015;7. 10.1186/s11689-015-9113-x. 10.1186/s11689-015-9113-x PMC4487986 26137170

[23] Olsen L, Sparsø T, Weinsheimer SM, Dos Santos MBQ, Mazin W, Rosengren A (2018). Prevalence of rearrangements in the 22q11.2 region and population-based risk of neuropsychiatric and developmental disorders in a Danish population: a case-cohort study. Lancet Psychiatry.

[24] McDonald-McGinn DM, Sullivan KE, Marino B, Philip N, Swillen A, Vorstman JAS, et al. 22Q11.2 Deletion Syndrome. Nat Rev Dis Prim. 2015;1:15071. 10.1038/nrdp.2015.71 PMC4900471 27189754

[25] Schneider M, Debbané M, Bassett AS, Chow EWC, Fung WLA, Van Den Bree MBM (2014). Psychiatric disorders from childhood to adulthood in 22q11.2 deletion syndrome: results from the international consortium on brain and behavior in 22q11.2 deletion syndrome. Am J Psychiatry.

[26] Malhotra D, Sebat J (2012). CNVs: Harbingers of a rare variant revolution in psychiatric genetics. Cell.

[27] Green T, Gothelf D, Glaser B, Debbane M, Frisch A, Kotler M (2009). Psychiatric disorders and intellectual functioning throughout development in velocardiofacial (22q11.2 deletion) syndrome. J Am Acad Child Adolesc Psychiatry.

[28] Chow EWC, Watson M, Young DA, Bassett AS (2006). Neurocognitive profile in 22q11 deletion syndrome and schizophrenia. Schizophr Res.

[29] Rees E, Kendall K, Europe PMC Funders Group. Analysis of intellectual disability copy number variants for association with schizophrenia. JAMA Psychiatry. 2017;73:963–9. 10.1001/jamapsychiatry.2016.1831 PMC5014093 27602560

[30] Marshall CR, Howrigan DP, Merico D, Thiruvahindrapuram B, Wu W, Greer DS (2017). Contribution of copy number variants to schizophrenia from a genome-wide study of 41,321 subjects. Nat Genet.

[31] Hemmings CP (2006). Schizophrenia spectrum disorders in people with intellectual disabilities. Curr Opin Psychiatry.

[32] Rees E, Kirov G, Sanders A, Walters JTR, Chambert KD, Shi J (2014). Evidence that duplications of 22q11.2 protect against schizophrenia. Mol Psychiatry.

[33] Li Z, Chen J, Xu Y, Yi Q, Ji W, Wang P (2016). Genome-wide analysis of the role of copy number variation in schizophrenia risk in Chinese. Biol Psychiatry.

[34] Lin A, Ching CRK, Vajdi A, Sun D, Jonas RK, Jalbrzikowski M (2017). Mapping 22q11.2 gene dosage effects on brain morphometry. J Neurosci.

[35] Lin A, Vajdi A, Kushan-Wells L, Helleman G, Hansen LP, Jonas RK (2020). Reciprocal copy number variations at 22q11.2 produce distinct and convergent neurobehavioral impairments relevant for schizophrenia and autism spectrum disorder. Biol Psychiatry.

[36] Yirmiya ET, Mekori-Domachevsky E, Weinberger R, Taler M, Carmel M, Gothelf D (2020). Exploring the potential association among sleep disturbances, cognitive impairments, and immune activation in 22q11.2 deletion syndrome. Am J Med Genet Part A.

[37] Moulding HA, Bartsch U, Hall J, Jones MW, Linden DE, Owen MJ, et al. Sleep problems and associations with psychopathology and cognition in young people with 22q11.2 deletion syndrome (22q11.2DS).Psychol Med. 2020;50:1191–202. 10.1017/S0033291719001119 31144615

[38] Arganbright JM, Tracy M, Hughes SS, Ingram DG (2020). Sleep patterns and problems among children with 22q11 deletion syndrome. Mol Genet Genomic Med.

[39] Hyde J, Eidels A, van Amelsvoort T, Myin-Germeys I, Campbell L. Gene deletion and sleep depletion: exploring the relationship between sleep and affect in 22q11.2 deletion syndrome. J Genet Psychol. 2021;182:304–16. 10.1080/00221325.2021.1930995 34114933

[40] Lunsford-Avery JR, Dean DJ, Mittal VA (2017). Self-reported sleep disturbances associated with procedural learning impairment in adolescents at ultra-high risk for psychosis. Schizophr Res.

[41] Lunsford-Avery JR, Orr JM, Gupta T, Pelletier-Baldelli A, Dean DJ, Smith Watts AK (2013). Sleep dysfunction and thalamic abnormalities in adolescents at ultra high-risk for psychosis. Schizophr Res.

[42] Malow BA, Marzec ML, McGrew SG, Wang L, Henderson LM, Stone WL (2006). Characterizing sleep in children with autism spectrum disorders: a multidimensional approach. Sleep..

[43] Jalbrzikowski M, Ahmed KH, Patel A, Jonas R, Chow C, Bearden CE (2018). Categorical versus dimensional approaches to autism-associated intermediate phenotypes in 22q11.2 microdeletion syndrome.

[44] Hundley RJ, Shui A, Malow BA (2016). Relationship between subtypes of restricted and repetitive behaviors and sleep disturbance in autism spectrum disorder. J Autism Dev Disord.

[45] Lunsford-Avery JR, LeBourgeois MK, Gupta T, Mittal VA (2015). Actigraphic-measured sleep disturbance predicts increased positive symptoms in adolescents at ultra high-risk for psychosis: a longitudinal study. Schizophr Res.

[46] Verhoeff ME, Blanken LME, Kocevska D, Mileva-Seitz VR, Jaddoe VWV, White T (2018). The bidirectional association between sleep problems and autism spectrum disorder: A population-based cohort study. Mol Autism.

[47] Miller TJ, McGlashan TH, Rosen JL, Cadenhead K, Cannon T, Ventura J (2004). Prodromal assessment with the structured interview for prodromal syndromes and the scale of prodromal symptoms: Predictive validity, interrater reliability, and training to reliability (Schizophrenia Bulletin (2003) 29, 4 (703-715)). Schizophr Bull.

[48] Schreck KA, Mulick JA, Smith AF (2004). Sleep problems as possible predictors of intensified symptoms of autism. Res Dev Disabil.

[49] Johnson CR, Smith T, DeMand A, Lecavalier L, Evans V, Gurka M (2018). Exploring sleep quality of young children with autism spectrum disorder and disruptive behaviors. Sleep Med.

[50] Lam KSL, Aman MG (2007). The repetitive behavior scale-revised: independent validation in individuals with autism spectrum disorders. J Autism Dev Disord.

[51] Constantino J, Gruber C (2005). Social Responsiveness Scale.

[52] Jalal R, Nair A, Lin A, Eckfeld A, Kushan L, Zinberg J (2021). Social cognition in 22q11.2 deletion syndrome and idiopathic developmental neuropsychiatric disorders. J Neurodev Disord.

[53] Gioia GA, Isquith PK, Guy SC, Kenworthy L, Baron IS (2000). Behavior rating inventory of executive function. Child Neuropsychol.

[54] Achenbach TM (1999). The Child Behavior Checklist and related instruments. use Psychol. Test. Treat. Plan. outcomes assessment.

[55] Achenbach TM, Rescorla LA (2001). Manual for the ASEBA school-age forms & profiles: child behavior checklist for ages 6-18, teacher’s report form, youth self-report: an integrated system of multi-informant assessment.

[56] Goldman SE, Surdyka K, Cuevas R, Adkins K, Wang L, Malow BA (2009). Defining the sleep phenotype in children with autism. Dev Neuropsychol.

[57] Becker SP, Ramsey RR, Byars KC (2015). Convergent validity of the Child Behavior Checklist sleep items with validated sleep measures and sleep disorder diagnoses in children and adolescents referred to a sleep disorders center. Sleep Med.

[58] Gregory AM, Cousins JC, Forbes EE, Trubnick L, Ryan ND, Axelson DA (2011). Sleep items in the child behavior checklist: a comparison with sleep diaries, actigraphy, and polysomnography. J Am Acad Child Adolesc Psychiatry.

[59] Goines KB, LoPilato AM, Addington J, Bearden CE, Cadenhead KS, Cannon TD (2019). Sleep problems and attenuated psychotic symptoms in youth at clinical high-risk for psychosis. Psychiatry Res.

[60] Grivel MM, Leong W, Masucci MD, Altschuler RA, Arndt LY, Redman SL (2018). Impact of lifetime traumatic experiences on suicidality and likelihood of conversion in a cohort of individuals at clinical high-risk for psychosis. Schizophr Res.

[61] Lindgren M, Manninen M, Kalska H, Mustonen U, Laajasalo T, Moilanen K, et al. Suicidality, self-harm and psychotic-like symptoms in a general adolescent psychiatric sample. Early Interv. Psychiatry. 2017;11:113–22. 10.1111/eip.12218 25582971

[62] R Core Team. R: A language and environment for statistical computing. R Foundation for Statistical Computing; 2021.

[63] Bates D, Mächler M, Bolker B, Walker S (2015). Fitting linear mixed-effects models using lme4. J Stat Softw.

[64] Wickham H (2016). ggplot2: elegant graphics for data analysis.

[65] Huhdanpää H, Klenberg L, Westerinen H, Fontell T, Aronen ET (2018). Sleep and psychiatric symptoms in young child psychiatric outpatients. Clin Child Psychol Psychiatry.

[66] Maurer GW, Malita A, Nagy S, Koyama T, Werge TM, Halberg KA, et al. Analysis of genes within the schizophrenia-linked 22q11.2 deletion identifies interaction of night owl/LZTR1 and NF1 in GABAergic sleep control. PLoS Genet. 2020;16:e1008727. 10.1371/journal.pgen.1008727 PMC7205319 32339168

[67] Hoftman GD, Volk DW, Bazmi HH, Li S, Sampson AR, Lewis DA (2015). Altered cortical expression of GABA-related genes in schizophrenia: Illness progression vs developmental disturbance. Schizophr Bull.

[68] Benes FM, Vincent SL, Alsterberg G, Bird ED, SanGiovanni JP (1992). Increased GABA(A) receptor binding in superficial layers of cingulate cortex in schizophrenics. J Neurosci.

[69] Schür RR, Draisma LWR, Wijnen JP, Boks MP, Koevoets MGJC, Joëls M (2016). Brain GABA levels across psychiatric disorders: a systematic literature review and meta-analysis of 1H-MRS studies. Hum Brain Mapp.

[70] Marsman A, Mandl RCW, Klomp DWJ, Bohlken MM, Boer VO, Andreychenko A (2014). GABA and glutamate in schizophrenia: A 7 T 1H-MRS study. NeuroImage Clin.

[71] Horder J, Petrinovic MM, Mendez MA, Bruns A, Takumi T, Spooren W (2018). Glutamate and GABA in autism spectrum disorder-a translational magnetic resonance spectroscopy study in man and rodent models. Transl Psychiatry.

[72] Kocevska D, Lysen TS, Dotinga A, Koopman-Verhoeff ME, Luijk MPCM, Antypa N (2021). Sleep characteristics across the lifespan in 1.1 million people from the Netherlands, United Kingdom and United States: a systematic review and meta-analysis. Nat Hum Behav.

[73] Hwang DK, Nam M, Lee YJG (2019). The effect of cognitive behavioral therapy for insomnia in schizophrenia patients with sleep disturbance: a non-randomized, assessor-blind trial. Psychiatry Res.

[74] Kennedy WP, Mudd PA, Maguire MA, Souders MC, McDonald-McGinn DM, Marcus CL (2014). 22q11.2 Deletion syndrome and obstructive sleep apnea. Int J Pediatr Otorhinolaryngol.

[75] Crockett DJ, Goudy SL, Chinnadurai S, Wootten CT (2014). Obstructive sleep apnea syndrome in children with 22q11.2 deletion syndrome after operative intervention for velopharyngeal insufficiency. Front Pediatr.

[76] Durdik P, Sujanska A, Suroviakova S, Evangelisti M, Banovcin P, Villa MP (2018). Sleep architecture in children with common phenotype of obstructive sleep apnea. J Clin Sleep Med.

[77] Reeves G, Blaisdell C, Lapidus M, Langenberg P, Ramagopal M, Cabassa J (2010). Sleep architecture and behavioral abnormalities in children and adolescents. Int J Adolesc Med Health.

