# Supplementary material for: Copy number variation at the 22q11.2 locus influences prevalence, severity, and psychiatric impact of sleep disturbance
Source: J Neurodev Disord. 2022 Jul 10;14:41. doi: 10.1186/s11689-022-09450-0 (PMC9275284; doi:10.1186/s11689-022-09450-0)
Supplement: Supplementary file 1 — Additional file 1: Supplementary Table 1. Summary of clinical measures, validated age range, age range of sample, reporter, and number of subjects who completed the measure. Supplementary Table 2. Scoring of Sleep Disturbance Item on SIPS. Supplementary Figure 1. Correlation between sleep items on SIPS and CBCL. Supplementary Table 3. Categorization of Participants According to Each Sleep Measure. Supplementary Figure 2. Summary scores of 22q11.2 CNV carriers who completed SIPS. Supplementary Figure 3. CBCL Scores of 22q11.2 CNV carriers who completed SIPS. Supplementary Table 4. Results of cross-sectional model including only participants who completed the SIPS sleep measure. Supplementary Table 5. Results of longitudinal models including only participants who completed SIPS sleep measure. Supplementary Table 6. Results of cross-sectional models. Supplementary Table 7. Results of longitudinal models. Supplementary Figure 4. SIPS subdomain scores in good and poor sleepers at each timepoint within 22q11.2 CNV groups. Supplementary Figure 5. RBS subdomain scores in good and poor sleepers at each timepoint within 22q11.2 CNV groups. Supplementary Figure 6. SRS subdomain scores in good and poor sleepers at each timepoint within 22q11.2 CNV groups. Supplementary Figure 7. BRIEF subdomain scores in good and poor sleepers at each timepoint within 22q11.2 CNV groups. [file 11689_2022_9450_MOESM1_ESM.docx]

***Supplementary Information***

**Supplementary Table 1.** Summary of clinical measures, validated age range, age range of sample, reporter, and number of subjects who completed the measure.

| **Measure** | **Validated Age Range** | **Age Range of Study Sample** | **Reporter** | **# of Subjects completing each measure** |
| --- | --- | --- | --- | --- |
| SIPS | 9-adult [1] | 9-49 | Clinician | 119 |
| RBS | 6-17 [2] | 5-21 | Parent | 113 |
| SRS | 2.5-18 [3] | 5-21 | Parent | 116 |
| BRIEF | 5-18 [4] | 5-21 | Parent | 109 |
| CBCL | 6-17 [5] | 5-22 | Parent | 104 |

**Validation of Categorical Sleep Variable**

Participants were categorized as good or poor sleepers based on their score for the sleep disturbance item on either the SIPs or the CBCL. On the SIPs item G1 (SIPSG1), a clinician rates levels of sleep disturbance on a scale from 0-6 based on participants’ reports of sleep difficulties and daytime functioning. The scoring of the SIPSG1 is displayed in Supplementary Table 1. Participants were considered a poor sleeper if they received a score of 3 or higher on the SIPS, which is considered to be clinically significant according to the SIPS scoring. Because the validity of the SIPS in young children has not been established, participants under 10 years of age did not complete the SIPS. For these participants, the sleep disturbance item on the CBCL (CBCL100) was used to classify them as good or poor sleepers. CBCL100 asks parents to rate their child’s trouble sleeping in the past six months from 0-2. A score of 0 indicates that it is not true that their child has trouble sleeping, a score of 1 indicates it is somewhat or sometimes true, and a score of 2 indicates that it is very true or often true that their child has trouble sleeping. Participants were considered to be poor sleepers if they received a rating of 2.

**Supplementary Table 2. Scoring of Sleep Disturbance Item on SIPS**

| **Score** | **Rating** | **Description** |
| --- | --- | --- |
| 0 | Absent |  |
| 1 | Questionably Present | Restless Sleep |
| 2 | Mild | Some mild difficulty falling asleep or getting back to sleep |
| 3 | Moderate | Daytime fatigue resulting from difficulty falling asleep at night or early awakening. Sleeping more than considered average |
| 4 | Moderately Severe | Sleep pattern significantly disrupted and has intruded other aspects of functioning. Difficult to awaken for appointments. Spending a large part of the day asleep. |
| 5 | Severe | Significant difficulty falling asleep or awakening early on most nights. May have day/night reversal. Usually not getting to scheduled activities at all. |
| 6 | Extreme | Unable to sleep for over 48 hours |

The SIPSG1 item is clinician report measure, while the CBCL100 is a parent-report measure. Due to this discrepancy and other differences across questionnaires, we calculated a Spearman Rank correlation coefficient between the two measures in the 54 22q11.2 CNV carriers who completed both measures to confirm concordance between the measures. We found the two measures were significantly positively correlated (*r*=0.493, p<0.001). Further, we compared the proportion of these 54 participants classified as poor sleepers between the two measures. The CBCL100 and SIPSG1 classified similar portions of the sample as poor sleepers (Supplementary Table 2.). These results led us to conclude that the summary categorical sleep variable is a valid measure in our sample.


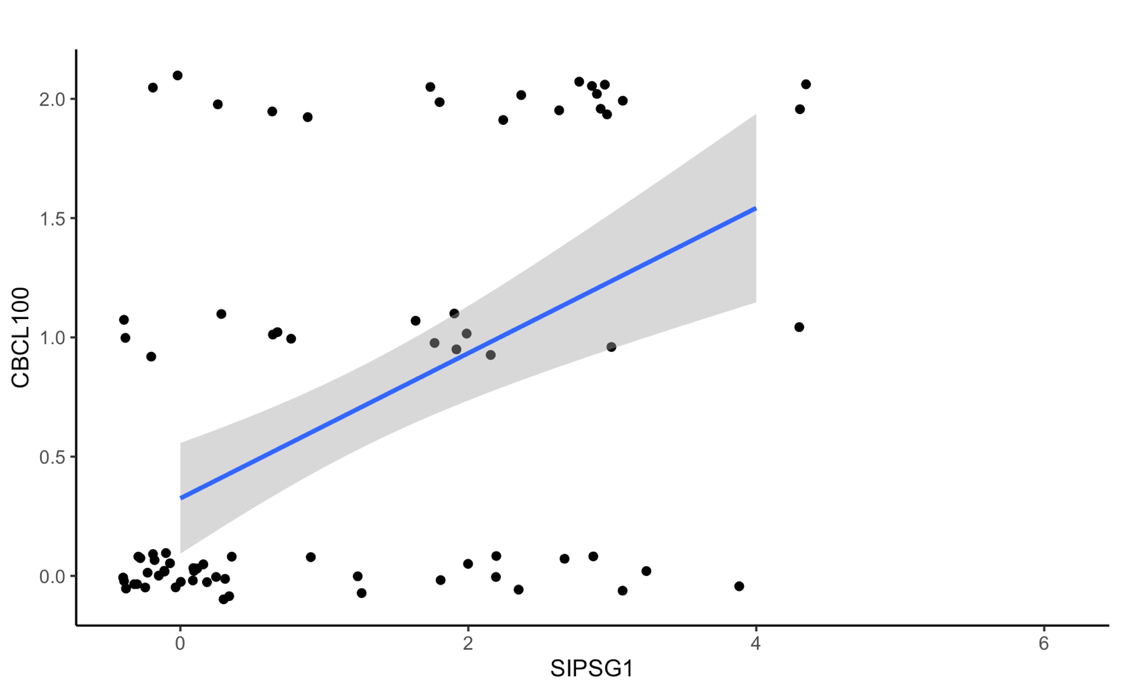


**Supplementary Figure 1. Correlation between sleep items on SIPS and CBCL.** There is a significant correlation between CBCL100 and SIPSG1scores at baseline in 22q11.2 carriers who completed both measures (n=54; r=0.493, p<0.001). Plot shows the jittered datapoints and regression line with the standard error.

**Supplementary Table 3.** Categorization of Participants According to Each Sleep Measure.

| n_total_=54 | Good Sleeper | Poor Sleeper |
| --- | --- | --- |
| CBCL100, *n (%)* | 38 (70.4%) | 16 (29.6%) |
| SIPSG1, *n (%)* | 40 (74.0%) | 14 (25.9%) |

****To confirm that the addition of younger participants who only completed the CBCL did not drive the direction of the effects observed, we re-ran the models only including CNV carriers who completed the SIPS. We tested the effect of the sleep category on the total summary scores for each measure. The uncorrected p-values are reported for cross-sectional models in Supplementary Table 4 and longitudinal models in Supplementary Table 5. As expected, results of these models were similar to the main analyses, but attenuated for all summary measures cross-sectionally and longitudinally, except RBS total score (Supplementary Figures 2 and 3). Surprisingly, removal of the younger subjects who did not complete the SIPS resulted in a significant group-by-sleep interaction and CNV group difference cross-sectionally, which was not present in the main analysis. The pattern of this interaction is consistent with other group-by-sleep interactions observed, such that there was a larger difference between good and poor sleepers in the 22qDel carriers compared to the 22qDup carriers.

**Supplementary Figure 2. Summary scores of 22q11.2 CNV carriers who completed SIPS**. Total RBS, SRS, and BRIEF global composite scores of 22q11.2 CNV carriers who completed the SIPS. As anticipated, effect of sleep on total RBS score, SRS score and BRIEF global composite scores showed similar trends as the models including all subjects, cross-sectionally and longitudinally. Just as in the main analysis, here was a significant effect of sleep on total RBS (*p*=0.001) and SRS (*p*=0.032) scores cross-sectionally and longitudinally (RBS: *p=*0.027; SRS: *p*=0.029), but no effect of sleep on BRIEF Global Composite score (cross-sectional: *p*= 0.100; longitudinal: *p*=0.112). Cross-sectionally, there was a significant effect of CNV group (*p*=0.005) and a group-by-sleep interaction (*p*=0.020) on total RBS score. Large dots represent the group mean and 95% confidence interval for good and poor sleepers at each timepoint.

**Supplementary Figure 3. CBCL Scores of 22q11.2 CNV carriers who completed SIPS**. As anticipated, the effect of sleep on CBCL scores showed similar trends as the models including all subjects cross-sectionally and longitudinally. Cross-sectionally, all subdomains remained significant (*p*<0.037) except for rule-breaking (*p*=0.097), which was attenuated to a significant trend due to decreased sample size. Longitudinally, the effect of sleep on anxious/depressed (*p*=0.018), thought problems (*p*<0.001), and aggressive behavior (*p*=0.004) remained significant. However, the effect of sleep on withdrawn/depressed (*p*=0.136), somatic complaints (*p*=0.050), social problems (*p*=0.113), attention problems (*p*=0.118), and rule-breaking (*p*=0.259) was attenuated. Longitudinally, there was a significant effect of CNV group on thought problems (*p*=0.024) and rule breaking (*p*=0.025). Large dots represent the group mean and 95% confidence interval for good and poor sleepers at each timepoint.

|  | **Effect of Sleep** | | **Effect of CNV Group** | | **Group*Sleep Interaction** | |
| --- | --- | --- | --- | --- | --- | --- |
|  | *β* | *p-value* | *β* | *p-value* | *β* | *p-value* |
| Total Repetitive Behaviors | **0.916** | **0.001** | **0.838** | **0.005** | -**1.204** | **0.020** |
| Social Responsiveness | **0.607** | **0.032** | 0.381 | 0.200 | -0.467 | 0.368 |
| BRIEF Global Composite | 0.497 | 0.100 | 0.234 | 0.443 | 0.022 | 0.968 |
| Anxious/Depressed | **0.655** | **0.030** | -0.169 | 0.592 | -0.740 | 0.189 |
| Withdrawn/Depressed | **0.688** | **0.021** | -0.078 | 0.801 | **-1.134** | **0.042** |
| Somatic Complaints | **0.798** | **0.008** | -0.021 | 0.947 | **-0.911** | **0.008** |
| Social Problems | **0.673** | **0.025** | 0.016 | 0.960 | -1.100 | 0.052 |
| Thought Problems | **1.120** | **<0.001** | -0.219 | 0.441 | -0.902 | 0.076 |
| Attention Problems | **0.626** | **0.036** | -0.098 | 0.754 | -1.027 | 0.061 |
| Rule Breaking | **0.479** | 0.097 | -0.272 | 0.371 | -1.254 | 0.097 |
| Aggressive Behavior | **1.120** | **<0.001** | 0.355 | 0.236 | **-1.547** | **0.005** |

**Supplementary Table 4.** Results of cross-sectional model including only participants who completed the SIPS sleep measure

**Supplementary Table 5.** Results of longitudinal models including only participants who completed SIPS sleep measure

|  | **Effect of Sleep** | | **Effect of CNV Group** | | **Effect of Time** | | **Group*Time Interaction** | |
| --- | --- | --- | --- | --- | --- | --- | --- | --- |
|  | *β* | *p-value* | *β* | *p-value* | *β* | *p-value* | *β* | *p-value* |
| Total Repetitive Behaviors | **0.521** | **0.027** | 0.476 | 0.061 | -0.097 | 0.273 | 0.062 | 0.693 |
| Social Responsiveness | **0.439** | **0.049** | 0.209 | 0.394 | -0.252 | 0.081 | **0.615** | **0.047** |
| BRIEF Global Composite | 0.359 | 0.112 | 0.223 | 0.371 | -0.198 | 0.206 | 0.053 | 0.856 |
| Anxious/Depressed | **0.538** | **0.018** | -0.431 | 0.094 | -0.222 | 0.280 | 0.372 | 0.346 |
| Withdrawn/Depressed | 0.336 | 0.136 | -0.461 | 0.076 | -0.343 | 0.116 | 0.396 | 0.345 |
| Somatic Complaints | 0.439 | 0.050 | -0.330 | 0.209 | -0.290 | 0.218 | 0.501 | 0.274 |
| Social Problems | 0.348 | 0.113 | -0.367 | 0.161 | -0.373 | 0.122 | -0.254 | 0.586 |
| Thought Problems | **0.954** | **<0.001** | **-0.539** | **0.024** | 0.213 | 0.301 | 0.211 | 0.595 |
| Attention Problems | 0.359 | 0.118 | -0.455 | 0.081 | -0.345 | 0.101 | 0.478 | 0.234 |
| Rule Breaking | 0.241 | 0.259 | **-0.582** | **0.025** | -0.185 | 0.442 | 0.017 | 0.972 |
| Aggressive Behavior | **0.645** | **0.004** | -0.147 | 0.561 | -0.340 | 0.408 | 0.340 | 0.408 |

**Results of SIPS Subscale Analysis**

Across CNV groups, poor sleepers reported more severe negative, disorganized, and general psychosis-risk symptoms than good sleepers, both cross-sectionally (Supplementary Table 6) and longitudinally (Supplementary Table 7) as shown in Supplementary Figure 4. 22qDel subjects reported higher severity of negative symptoms, both cross-sectionally and longitudinally, and a higher severity of disorganized psychosis-risk symptoms longitudinally compared to 22qDup.

**Results of RBS and SRS Subscales Analysis**

Poor sleepers, across CNV groups, reported more frequent self-injurious, restrictive, compulsive, and ritualistic behaviors on the RBS at baseline (Supplementary Table 6) and across timepoints (Supplementary Table 7; Supplementary Figure 5). Poor sleepers reported increased frequency of need for sameness behaviors at baseline only. There were no statistically significant effects of sleep on the stereotyped behaviors subscales. Cross-sectionally across CNV groups, poor sleep scored higher (i.e., more pathological) on the SRS communication subscale (Supplementary Figure 6). Longitudinally, poor sleepers scored significantly higher on the aware, cognition, communication, and autistic traits subscales of the SRS. There was no significant effect of sleep on the motivation subscale.

**Results of BRIEF Subscale Analysis**

There was a significant main effect of sleep categorization, in which poor sleepers scored higher (i.e. more pathological), on the shift, emotional control, initiate subscales cross-sectionally (Supplementary Figure 7; Supplementary Table 6). Longitudinally, poor sleepers scored higher on shifting, emotional control, and monitoring subscales (Supplementary Table 7). No other effects of sleep survived FDR correction.

**Supplementary Table 6.** Results of cross-sectional models

|  | **Effect of Sleep** | | **Effect of CNV Group** | | **Group*Sleep Interaction** | |
| --- | --- | --- | --- | --- | --- | --- |
| **Psychosis-Risk Symptoms (SIPS)** | *β* | *q-value* | *β* | *q-value* | *β* | *q-value* |
| Negative Symptoms | **0.502** | **0.041** | **-0.659** | **0.028** | -0.223 | 0.570 |
| General Symptoms | **0.860** | **<0.001** | -0.358 | 0.180 | -0.505 | 0.234 |
| Disorganized Symptoms | **0.838** | **<0.001** | -0.040 | 0.871 | -0.546 | 0.234 |
| **Repetitive Behavior Scale (RBS)** | *β* | *q-value* | *β* | *q-value* | *β* | *q-value* |
| Stereotyped Behaviors | 0.120 | 0.607 | **0.691** | **0.008** | 0.283 | 0.607 |
| Self-Injurious Behaviors | **0.844** | **0.002** | 0.113 | 0.630 | -0.272 | 0.630 |
| Compulsive Behaviors | **0.716** | **0.013** | 0.098 | 0.682 | -0.282 | 0.682 |
| Ritualistic Behavior | **0.642** | **0.032** | 0.265 | 0.404 | -0.156 | 0.732 |
| Sameness Behavior | **0.712** | **0.014** | 0.418 | 0.083 | -0.832 | 0.083 |
| Restrictive Behavior | **0.724** | **0.011** | 0.195 | 0.412 | -0.705 | 0.120 |
| **Social Responsiveness Scale (SRS)** | *β* | *q-value* | *β* | *q-value* | *β* | *q-value* |
| Aware | 0.561 | 0.085 | 0.171 | 0.567 | -0.266 | 0.567 |
| Cognition | 0.553 | 0.087 | 0.149 | 0.534 | -0.293 | 0.534 |
| Communication | **0.640** | **0.029** | 0.312 | 0.272 | -0.171 | 0.701 |
| Motivation | 0.579 | 0.058 | 0.150 | 0.518 | -0.476 | 0.432 |
| Autistic Traits | 0.531 | 0.099 | 0.224 | 0.513 | 0.059 | 0.897 |
| **Real-World Executive Function (BRIEF)** | *β* | *q-value* | *β* | *q-value* | *β* | *q-value* |
| Inhibition | 0.413 | 0.225 | 0.334 | 0.225 | 0.129 | 0.768 |
| Shifting | **0.662** | **0.023** | 0.300 | 0.316 | -0.066 | 0.883 |
| Emotional | **0.726** | **0.008** | 0.298 | 0.301 | -0.095 | 0.830 |
| Initiate | **0.673** | **0.016** | 0.354 | 0.192 | -0.005 | 0.991 |
| Working Memory | 0.329 | 0.267 | 0.206 | 0.387 | -0.719 | 0.267 |
| Plan/Organize | 0.497 | 0.142 | -0.121 | 0.888 | 0.064 | 0.888 |
| Organization/Materials | 0.349 | 0.520 | -0.105 | 0.845 | -0.091 | 0.845 |
| Monitor | 0.210 | 0.996 | -0.001 | 0.996 | 0.130 | 0.996 |

|  | **Effect of Sleep** | | **Effect of CNV Group** | | **Effect of Time** | | **Group*Time Interaction** | |
| --- | --- | --- | --- | --- | --- | --- | --- | --- |
| **Psychosis-Risk Symptoms** | *β* | *q-value* | *β* | *q-value* | *β* | *p-value* | *β* | *p-value* |
| Negative Symptoms | **0.426** | **0.019** | **-0.734** | **<0.001** | -0.119 | 0.277 | **0.574** | **0.009** |
| General Symptoms | **0.710** | **<0.001** | -0.245 | 0.203 | -0.186 | 0.164 | -0.051 | 0.844 |
| Disorganized Symptoms | **0.575** | **0.003** | **-0.540** | **0.009** | -0.054 | 0.631 | 0.160 | 0.473 |
| **Repetitive Behavior Scale (RBS)** | *β* | *q-value* | *β* | *q-value* | *β* | *p-value* | *β* | *p-value* |
| Stereotyped Behaviors | 0.167 | 0.345 | **0.736** | **<0.001** | 0.056 | 0.724 | -0.498 | 0.120 |
| Self-Injurious Behaviors | **0.489** | **0.025** | 0.027 | 0.896 | 0.043 | 0.764 | 0.123 | 0.664 |
| Compulsive Behaviors | **0.529** | **0.020** | 0.013 | 0.951 | 0.006 | 0.963 | -0.072 | 0.792 |
| Ritualistic Behavior | **0.591** | **0.006** | 0.225 | 0.800 | 0.011 | 0.937 | -0.047 | 0.937 |
| Sameness Behavior | 0.357 | 0.164 | 0.195 | 0.362 | -0.158 | 0.232 | 0.197 | 0.454 |
| Restrictive Behavior | **0.477** | **0.034** | 0.002 | 0.994 | -0.053 | 0.704 | 0.078 | 0.781 |
| **Social Responsiveness Scale (SRS)** | *β* | *q-value* | *β* | *q-value* | *β* | *p-value* | *β* | *p-value* |
| Aware | **0.499** | **0.029** | 0.065 | 0.757 | 0.112 | 0.391 | 0.191 | 0.467 |
| Cognition | **0.464** | **0.047** | -0.048 | 0.820 | -0.150 | 0.225 | 0.237 | 0.340 |
| Communication | **0.534** | **0.018** | 0.250 | 0.223 | -0.163 | 0.131 | 0.189 | 0.376 |
| Motivation | 0.332 | 0.174 | 0.038 | 0.848 | -0.278 | 0.027 | 0.483 | 0.057 |
| Autistic Traits | **0.554** | **0.013** | 0.207 | 0.313 | -0.022 | 0.836 | 0.112 | 0.590 |
| **Real-World Executive Function (BRIEF)** | *β* | *q-value* | *β* | *q-value* | *β* | *p-value* | *β* | *p-value* |
| Inhibition | 0.392 | 0.072 | 0.354 | 0.225 | -0.032 | 0.739 | 0.194 | 0.311 |
| Shifting | **0.519** | **0.017** | 0.300 | 0.146 | -0.132 | 0.307 | 0.298 | 0.253 |
| Emotional | **0.644** | **0.002** | 0.260 | 0.180 | **-0.285** | **0.006** | 0.343 | 0.089 |
| Initiate | 0.032 | 0.990 | 0.003 | 0.990 | -0.273 | 0.084 | 0.056 | 0.859 |
| Working Memory | 0.411 | 0.071 | 0.343 | 0.635 | -0.323 | 0.017 | 0.343 | 0.204 |
| Plan/Organize | 0.237 | 0.464 | -0.157 | 0.466 | -0.199 | 0.168 | 0.461 | 0.117 |
| Organization/Materials | 0.188 | 0.688 | 0.018 | 0.929 | -0.159 | 0.160 | 0.222 | 0.160 |
| Monitor | **0.442** | **0.044** | 0.083 | 0.680 | **-0.352** | **0.005** | **0.625** | **0.012** |

**Supplementary Table 7.** Results of longitudinal models

**
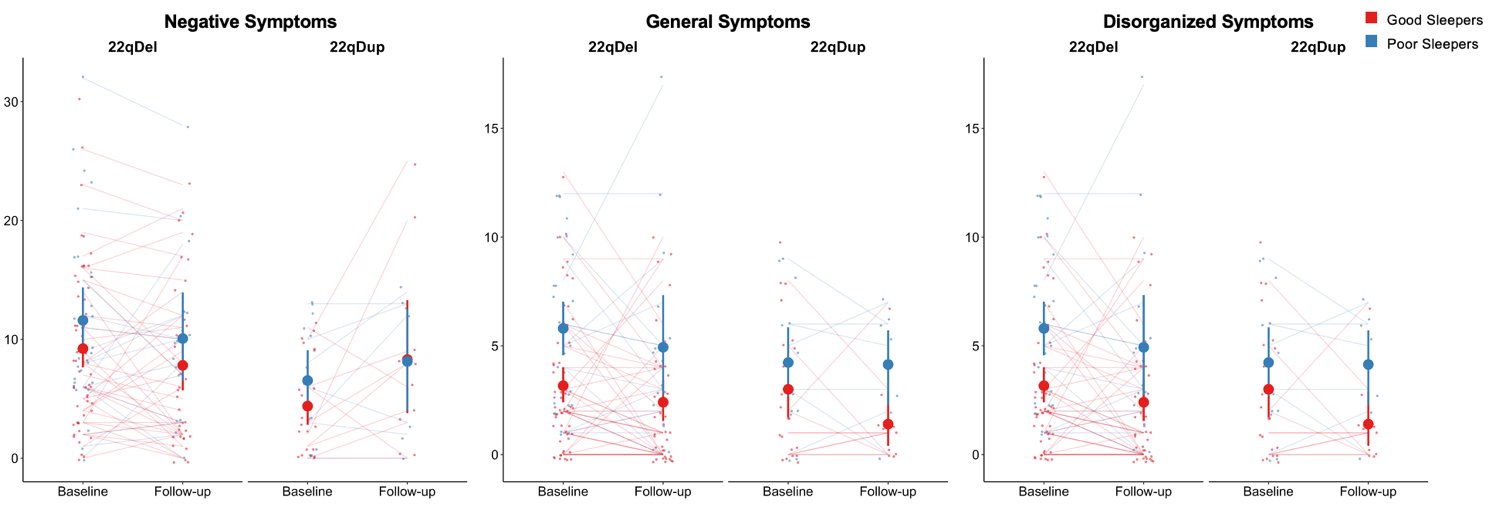
Supplementary Figure 4. SIPS subdomain scores in good and poor sleepers at each timepoint within 22q11.2 CNV groups.** There was a significant main effect of sleep category on negative (*q*<0.042), disorganized (*q*<0.003), and general symptoms (*q*<0.001) cross-sectionally and longitudinally. Large dots represent the group mean and 95% confidence interval for good and poor sleepers at each timepoint.

**Supplementary Figure 5. RBS subdomain scores in good and poor sleepers at each timepoint within 22q11.2 CNV groups**. At baseline, there was a significant main effect of sleep category on self-injurious (*q*=0.002), ritualistic (*q=*0.032), need for sameness (*q=*0.014), and restrictive behaviors (*q=*0.011). Across timepoints, there was a significant main effect of sleep category on self-injurious (*q*=0.025), compulsive (*q*=0.020), ritualistic (*q*=0.006), and restrictive behaviors (*q*=0.034 ). Large dots represent the group mean and 95% confidence interval for good and poor sleepers at each timepoint.

**Supplementary Figure 6. SRS subdomain scores in good and poor sleepers at each timepoint within 22q11.2 CNV groups**. There was a significant main effects of sleep category on the communication subscale at baseline (*q*= 0.029). Longitudinally, there was a significant main effect of sleep category on aware (*q*=0.029), cognition (*q*=0.047), communication (*q*=0.018), and autistic traits (*q*=0.013) subscales. There was no significant effect of sleep category on the motivation subscale (*q*=0.174). Large dots represent the group mean and 95% confidence interval for good and poor sleepers at each timepoint.

**Supplementary Figure 7. BRIEF subdomain scores in good and poor sleepers at each timepoint within 22q11.2 CNV groups**. At baseline, there poor sleepers scored worse on shifting (*q*=0.029), emotional (*q=*0.047), and initiate (*q*=0.018*)* subscales. Longitudinally, there was a significant main effect of sleep category on shifting (*q*=0.017), emotional (*q*=0.002), and monitor (*q*=0.044) subscales. Large dots represent the group mean and 95% confidence interval for good and poor sleepers at each timepoint.

**Sleep Apnea Considerations**

22q11.2 CNVs are associated with craniofacial abnormalities, which lead to increased rates of sleep-disordered breathing (SDB) among this population [6,7]. Sleep disturbance related to SDB confers a different clinical phenotype and affects downstream processes differently than a sleep disturbance related to insufficient sleep [8,9]. SDB was not directly measured in this study and it often goes undiagnosed, so to investigate if the high rates of poor sleepers present in the 22q11.2 CNV groups is accounted for by increased SDB, we compared the rates of good and poor sleepers that reported a craniofacial abnormality and the rates who reported a medical condition related to breathing. Within the CNV carriers, 72% of poor sleepers and 62% of good sleepers reported a craniofacial abnormality. 84% of poor sleepers and 81% of good sleepers reported a breathing-related medical condition. While the proportions are slightly higher in the poor sleeper group, the difference was not statistically significant for craniofacial abnormalities (X^2^=0.403; *p*=0.525), nor breathing conditions (X^2^=0.005; *p*=0.942). Thus, it is unlikely that SDB plays a large role in the sleep disturbance differences observed.

**References**

1. Calkins ME, Merikangas KR, Moore TM, Burstein M, Behr MA, Satterthwaite TD, et al. The Philadelphia neurodevelopmental cohort: Constructing a deep phenotyping collaborative. J. Child Psychol. Psychiatry. Calkins, Monica E.: Department of Psychiatry, Neuropsychiatry Section, Perelman School of Medicine, University of Pennsylvania, 9 Maloney, 3600 Spruce Street, Philadelphia, PA, US, 19104, mcalkins@upenn.edu: Wiley-Blackwell Publishing Ltd.; 2015. p. 1356–69.

2. Lam KSL, Aman MG. The Repetitive Behavior Scale-Revised: Independent Validation in Individuals with Autism Spectrum Disorders. J Autism Dev Disord [Internet]. 2007;37:855–66. Available from: https://doi.org/10.1007/s10803-006-0213-z

3. Constantino J, Gruber C. Social Responsiveness Scale 2nd ed: Western Psychological Services. 2005;

4. Gioia GA, Isquith PK, Guy SC, Kenworthy L, Baron IS. Behavior rating inventory of executive function. Child Neuropsychol. 2000;6:235–8.

5. Achenbach TM. The Child Behavior Checklist and related instruments. use Psychol. Test. Treat. Plan. outcomes assessment, 2nd ed. Mahwah, NJ, US: Lawrence Erlbaum Associates Publishers; 1999. p. 429–66.

6. Kennedy WP, Mudd PA, Maguire MA, Souders MC, McDonald-McGinn DM, Marcus CL, et al. 22q11.2 Deletion syndrome and obstructive sleep apnea. Int J Pediatr Otorhinolaryngol [Internet]. 2014;78:1360–4. Available from: https://www.sciencedirect.com/science/article/pii/S0165587614003164

7. Crockett DJ, Goudy SL, Chinnadurai S, Wootten CT. Obstructive sleep apnea syndrome in children with 22q11.2 deletion syndrome after operative intervention for velopharyngeal insufficiency. Front Pediatr. 2014;2:1–5.

8. Durdik P, Sujanska A, Suroviakova S, Evangelisti M, Banovcin P, Villa MP. Sleep architecture in children with common phenotype of obstructive sleep apnea. J Clin Sleep Med. 2018;14:9–14.

9. Reeves G, Blaisdell C, Lapidus M, Langenberg P, Ramagopal M, Cabassa J, et al. Sleep architecture and behavioral abnormalities in children and adolescents. Int J Adolesc Med Health. 2010;22:535–45.
